# Ethyl 7-pivaloyl­amino-1,8-naphthyridine-2-carboxyl­ate sesquihydrate

**DOI:** 10.1107/S160053681104921X

**Published:** 2011-11-25

**Authors:** Hoong-Kun Fun, Madhukar Hemamalini, Anita Hazra, Shyamaprosad Goswami

**Affiliations:** a X-ray Crystallography Unit, School of Physics, Universiti Sains Malaysia, 11800 USM, Penang, Malaysia; b Department of Chemistry, Bengal Engineering and Science University, Shibpur, Howrah 711 103, India

## Abstract

In the title hydrate, C_16_H_19_N_3_O_3_·1.5H_2_O, both water mol­ecules are disordered: one over two adjacent sites in a 0.498 (5):0.502 (5) ratio and one lying near a crystallographic twofold axis. The dihedral angle between the pyridine rings of the organic moleucle is 1.47 (6)°. In the crystal, the components are linked by N—H⋯O, O—H⋯N and C—H⋯O hydrogen bonds, forming sheets lying parallel to the *ac* plane.

## Related literature

For further details of heterocyclic esters, see: Listvan *et al.* (2002 ▶); Li *et al.* (2007 ▶); Goswami & Hazra (2009 ▶). For the stability of the temperature controller used in the data collection, see: Cosier & Glazer (1986 ▶).
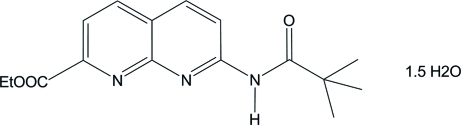


## Experimental

### 

#### Crystal data

C_16_H_19_N_3_O_3_·1.5H_2_O
*M* *_r_* = 656.73
Monoclinic, 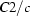

*a* = 30.7759 (7) Å
*b* = 7.2406 (2) Å
*c* = 16.9271 (4) Å
β = 120.009 (1)°
*V* = 3266.32 (14) Å^3^
*Z* = 4
Mo *K*α radiation
μ = 0.10 mm^−1^
*T* = 100 K
0.41 × 0.31 × 0.24 mm

#### Data collection

Bruker SMART APEXII CCD diffractometer
Absorption correction: multi-scan (*SADABS*; Bruker, 2009 ▶) *T*_min_ = 0.960, *T*_max_ = 0.977
16624 measured reflections
3753 independent reflections
3156 reflections with *I* > 2σ(*I*)
*R*_int_ = 0.030

#### Refinement

*R*[*F* ^2^ > 2σ(*F* ^2^)] = 0.039
*wR*(*F* ^2^) = 0.108
*S* = 1.04
3753 reflections
228 parameters
H-atom parameters constrained
Δρ_max_ = 0.34 e Å^−3^
Δρ_min_ = −0.23 e Å^−3^

### 

Data collection: *APEX2* (Bruker, 2009 ▶); cell refinement: *SAINT* (Bruker, 2009 ▶); data reduction: *SAINT*; program(s) used to solve structure: *SHELXTL* (Sheldrick, 2008 ▶); program(s) used to refine structure: *SHELXTL*; molecular graphics: *SHELXTL*; software used to prepare material for publication: *SHELXTL* and *PLATON* (Spek, 2009 ▶).

## Supplementary Material

Crystal structure: contains datablock(s) global, I. DOI: 10.1107/S160053681104921X/hb6505sup1.cif

Structure factors: contains datablock(s) I. DOI: 10.1107/S160053681104921X/hb6505Isup2.hkl

Supplementary material file. DOI: 10.1107/S160053681104921X/hb6505Isup3.cml

Additional supplementary materials:  crystallographic information; 3D view; checkCIF report

## Figures and Tables

**Table 1:** Hydrogen-bond geometry (Å, °)

*D*—H⋯*A*	*D*—H	H⋯*A*	*D*⋯*A*	*D*—H⋯*A*
N3—H1*N*3⋯O2*W*	0.85	2.39	3.051 (2)	135
N3—H1*N*3⋯O1*WB*^i^	0.85	2.40	3.095 (3)	140
O1*WB*—H2*WB*⋯N2	0.86	2.26	3.077 (3)	160
O2*W*—H1*W*2⋯N1^i^	0.83	2.13	2.948 (2)	167
C3—H3*A*⋯O3	0.93	2.23	2.8230 (17)	121

Symmetry code: (i) 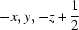.

**Fig. 1.**
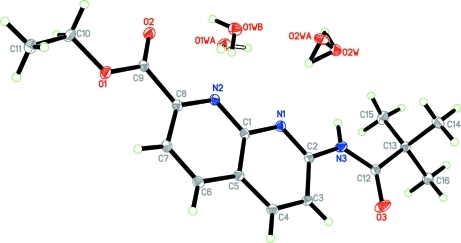
The asymmetric unit of the title compound, showing 30% probability displacement ellipsoids. Open bonds reprents disorder components.

**Fig. 2.**
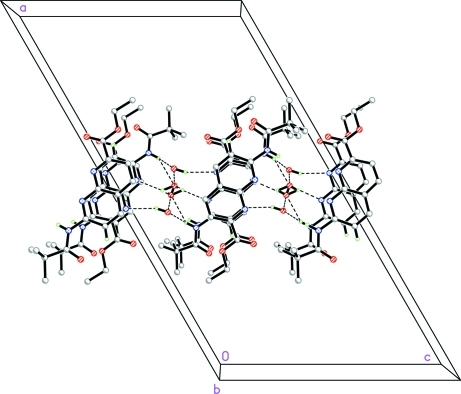
The crystal packing of the title compound (I). H atoms are not involing the hydrogen bond interactions are omitted for clarity.

## supplementary crystallographic information

### Comment

Heterocyclic esters are important synthons for the synthesis of different natural products, antimicrobial agents and pharmaceutical compositions (Listvan *et al.*, 2002; Li *et al.*, 2007). The heterocyclic esters are easily synthesized from their corresponding aldehydes by using thiamine hydrochloride as a catalyst in the presence of triethyl amine and alcohol (Goswami & Hazra, 2009).  Herein we report the crystal structure of ethyl-7-pivaloylamino-[1,8]naphthyridine-2-carboxylate.

The asymmetric unit of the title compound, Fig. 1, consists of one ethyl-7-pivaloylamino-[1,8]naphthyridine-2-carboxylate molecule, one disordered water molecule over two orientations with a refined occupany ratio of 0.498 (5) : 0.502 (5) and a half-molecule of water (the O2W atom of the water molecule lies near a twofold axis (symmetry code: -x, y, -z+1/2).  The dihedral angle between the two pyridine (N1/C1–C5 : N2/C1,C5–C8) rings is 1.47 (6)°.

In the crystal structure, (Fig. 2), the components are connected *via* intermolecular N—H···O, O—H···N and C—H···O hydrogen bonds (Table 1) to form two-dimensional networks parallel to the *ac*-plane.

### Experimental

Distilled triethylamine (0.6 ml) was added dropwise to a solution of 7-pivaloylamino-[1,8]naphthyridine-2-carbaldehyde (514 mg, 2 mmol) in dry ethanol. Then thiamine hydrochloride (30 mg, 15 mol) was added and the reaction mixture was refluxed for 2.5 h. Excess ethanol was distilled from the reaction mixture after completion of the reaction. Water was added to the reaction mixture and then extracted with chloroform and the organic layer was dried. The crude product was purified through column chromatography (silica gel, 100–200 mesh) eluting with ethyl acetate in petroleum ether (30%) to afford a colorless solid.  Yield: 82%. Mp 168–170°C.

### Refinement

All hydrogen atoms were positioned geometrically [N–H = 0.8462 Å; O–H = 0.8078–0.9888 Å; C–H = 0.93–0.97 Å] and were refined using a riding model, with *U*_iso_(H) = 1.2 or 1.5 *U*_eq_(C,O). A rotating group model was applied to the methyl groups.  One of the water molecule is disordered over two orientations, with an occupany ratio of 0.498 (5) : 0.502 (5). Another water molecule, 02W, lies near a twofold axis with symmetry -x, y, -z+1/2 .

### Crystal data

**Table:**

C_16_H_19_N_3_O_3_·1.5H_2_O	*F*(000) = 1400
*M**_r_* = 656.73	*D*_x_ = 1.335 Mg m^−^^3^
Monoclinic, *C*2/*c*	Mo *K*α radiation, λ = 0.71073 Å
Hall symbol: -C 2yc	Cell parameters from 6305 reflections
*a* = 30.7759 (7) Å	θ = 2.9–33.5°
*b* = 7.2406 (2) Å	µ = 0.10 mm^−^^1^
*c* = 16.9271 (4) Å	*T* = 100 K
β = 120.009 (1)°	Block, colourless
*V* = 3266.32 (14) Å^3^	0.41 × 0.31 × 0.24 mm
*Z* = 4	

### Data collection

**Table:**

Bruker SMART APEXII CCD diffractometer	3753 independent reflections
Radiation source: fine-focus sealed tube	3156 reflections with *I* > 2σ(*I*)
graphite	*R*_int_ = 0.030
φ and ω scans	θ_max_ = 27.5°, θ_min_ = 2.9°
Absorption correction: multi-scan (*SADABS*; Bruker, 2009)	*h* = −39→39
*T*_min_ = 0.960, *T*_max_ = 0.977	*k* = −9→9
16624 measured reflections	*l* = −21→21

### Refinement

**Table:**

Refinement on *F*^2^	Primary atom site location: structure-invariant direct methods
Least-squares matrix: full	Secondary atom site location: difference Fourier map
*R*[*F*^2^ > 2σ(*F*^2^)] = 0.039	Hydrogen site location: inferred from neighbouring sites
*wR*(*F*^2^) = 0.108	H-atom parameters constrained
*S* = 1.04	*w* = 1/[σ^2^(*F*_o_^2^) + (0.0531*P*)^2^ + 2.3589*P*] where *P* = (*F*_o_^2^ + 2*F*_c_^2^)/3
3753 reflections	(Δ/σ)_max_ < 0.001
228 parameters	Δρ_max_ = 0.34 e Å^−^^3^
0 restraints	Δρ_min_ = −0.23 e Å^−^^3^

### Special details

**Table:**

Experimental. The crystal was placed in the cold stream of an Oxford Cryosystems Cobra open-flow nitrogen cryostat (Cosier & Glazer, 1986) operating at 100.0 (1) K.
Geometry. All s.u.'s (except the s.u. in the dihedral angle between two l.s. planes) are estimated using the full covariance matrix. The cell s.u.'s are taken into account individually in the estimation of s.u.'s in distances, angles and torsion angles; correlations between s.u.'s in cell parameters are only used when they are defined by crystal symmetry. An approximate (isotropic) treatment of cell s.u.'s is used for estimating s.u.'s involving l.s. planes.
Refinement. Refinement of F^2^ against ALL reflections. The weighted R-factor wR and goodness of fit S are based on F^2^, conventional R-factors R are based on F, with F set to zero for negative F^2^. The threshold expression of F^2^ > 2σ(F^2^) is used only for calculating R-factors(gt) etc. and is not relevant to the choice of reflections for refinement. R-factors based on F^2^ are statistically about twice as large as those based on F, and R- factors based on ALL data will be even larger.

### Fractional atomic coordinates and isotropic or equivalent isotropic displacement parameters (Å^2^)

**Table:**

	*x*	*y*	*z*	*U*_iso_*/*U*_eq_	Occ. (<1)
O1	0.16006 (3)	0.48093 (13)	0.67950 (6)	0.0212 (2)	
O2	0.14079 (3)	0.47791 (13)	0.53237 (6)	0.0232 (2)	
O3	−0.17328 (4)	−0.00262 (19)	0.24944 (7)	0.0422 (3)	
N1	−0.02737 (4)	0.20833 (14)	0.37662 (7)	0.0172 (2)	
N2	0.04907 (4)	0.32379 (14)	0.48603 (7)	0.0166 (2)	
N3	−0.10204 (4)	0.10828 (17)	0.25947 (7)	0.0245 (3)	
H1N3	−0.0879	0.1349	0.2290	0.029*	
C1	0.00362 (4)	0.25427 (15)	0.46599 (8)	0.0154 (2)	
C2	−0.07277 (4)	0.14680 (16)	0.35245 (8)	0.0173 (2)	
C3	−0.09081 (4)	0.12125 (17)	0.41469 (8)	0.0186 (2)	
H3A	−0.1231	0.0774	0.3947	0.022*	
C4	−0.05950 (4)	0.16280 (17)	0.50400 (8)	0.0186 (2)	
H4A	−0.0701	0.1455	0.5462	0.022*	
C5	−0.01072 (4)	0.23240 (16)	0.53302 (8)	0.0161 (2)	
C6	0.02364 (4)	0.28315 (17)	0.62367 (8)	0.0187 (2)	
H6A	0.0154	0.2698	0.6693	0.022*	
C7	0.06941 (4)	0.35249 (16)	0.64349 (8)	0.0182 (2)	
H7A	0.0928	0.3873	0.7027	0.022*	
C8	0.08024 (4)	0.36993 (15)	0.57230 (8)	0.0160 (2)	
C9	0.12989 (4)	0.44831 (16)	0.59031 (8)	0.0171 (2)	
C10	0.20924 (4)	0.55545 (19)	0.70542 (9)	0.0232 (3)	
H10A	0.2061	0.6797	0.6817	0.028*	
H10B	0.2258	0.4789	0.6813	0.028*	
C11	0.23881 (5)	0.5572 (2)	0.80776 (9)	0.0268 (3)	
H11A	0.2718	0.6046	0.8277	0.040*	
H11B	0.2413	0.4337	0.8303	0.040*	
H11C	0.2222	0.6343	0.8307	0.040*	
C12	−0.14904 (4)	0.03205 (16)	0.21292 (8)	0.0180 (2)	
C13	−0.16825 (4)	−0.01579 (17)	0.11242 (8)	0.0194 (3)	
C14	−0.14824 (5)	−0.20988 (19)	0.11108 (9)	0.0266 (3)	
H14A	−0.1603	−0.2964	0.1386	0.040*	
H14B	−0.1122	−0.2084	0.1447	0.040*	
H14C	−0.1597	−0.2459	0.0492	0.040*	
C15	−0.15098 (5)	0.1224 (2)	0.06522 (9)	0.0285 (3)	
H15A	−0.1599	0.2452	0.0732	0.043*	
H15B	−0.1669	0.0943	0.0013	0.043*	
H15C	−0.1152	0.1144	0.0916	0.043*	
C16	−0.22570 (5)	−0.02054 (19)	0.06234 (9)	0.0243 (3)	
H16A	−0.2382	0.0996	0.0645	0.037*	
H16B	−0.2366	−0.1086	0.0912	0.037*	
H16C	−0.2383	−0.0557	−0.0001	0.037*	
O1WB	0.05785 (9)	0.3977 (4)	0.31552 (15)	0.0289 (7)	0.502 (5)
H1WB	0.0755	0.4935	0.3217	0.035*	0.502 (5)
H2WB	0.0480	0.3869	0.3546	0.035*	0.502 (5)
O1WA	0.04715 (6)	0.4721 (2)	0.32308 (11)	0.0310 (7)	0.498 (5)
H1WA	0.0227	0.4263	0.2743	0.037*	0.498 (5)
H2WA	0.0604	0.4032	0.3761	0.037*	0.498 (5)
O2W	−0.01311 (6)	0.0366 (2)	0.23101 (11)	0.0245 (5)	0.50
H1W2	−0.0004	0.0990	0.2065	0.029*	

### Atomic displacement parameters (Å^2^)

**Table:**

	*U*^11^	*U*^22^	*U*^33^	*U*^12^	*U*^13^	*U*^23^
O1	0.0167 (4)	0.0275 (5)	0.0172 (4)	−0.0053 (3)	0.0067 (4)	−0.0017 (4)
O2	0.0228 (4)	0.0271 (5)	0.0213 (5)	−0.0056 (4)	0.0122 (4)	−0.0029 (4)
O3	0.0269 (5)	0.0798 (9)	0.0222 (5)	−0.0244 (5)	0.0140 (4)	−0.0140 (5)
N1	0.0174 (5)	0.0176 (5)	0.0162 (5)	−0.0011 (4)	0.0081 (4)	−0.0005 (4)
N2	0.0170 (5)	0.0149 (5)	0.0173 (5)	−0.0006 (4)	0.0081 (4)	−0.0004 (4)
N3	0.0195 (5)	0.0393 (7)	0.0165 (5)	−0.0083 (5)	0.0104 (4)	−0.0046 (5)
C1	0.0174 (5)	0.0120 (5)	0.0167 (6)	0.0013 (4)	0.0084 (5)	0.0010 (4)
C2	0.0178 (5)	0.0163 (5)	0.0172 (6)	0.0003 (4)	0.0082 (5)	−0.0010 (5)
C3	0.0164 (5)	0.0189 (6)	0.0218 (6)	−0.0005 (4)	0.0104 (5)	−0.0006 (5)
C4	0.0202 (6)	0.0192 (6)	0.0199 (6)	−0.0002 (5)	0.0126 (5)	0.0005 (5)
C5	0.0174 (5)	0.0143 (5)	0.0171 (6)	0.0013 (4)	0.0089 (5)	0.0004 (4)
C6	0.0220 (6)	0.0196 (6)	0.0160 (6)	0.0004 (5)	0.0107 (5)	0.0005 (5)
C7	0.0189 (5)	0.0176 (6)	0.0153 (6)	0.0000 (4)	0.0065 (5)	−0.0006 (5)
C8	0.0172 (5)	0.0119 (5)	0.0176 (6)	0.0013 (4)	0.0077 (5)	0.0004 (4)
C9	0.0188 (5)	0.0134 (5)	0.0181 (6)	0.0006 (4)	0.0084 (5)	−0.0004 (4)
C10	0.0164 (6)	0.0280 (7)	0.0234 (7)	−0.0057 (5)	0.0085 (5)	−0.0015 (5)
C11	0.0202 (6)	0.0318 (7)	0.0237 (7)	−0.0012 (5)	0.0074 (5)	−0.0044 (6)
C12	0.0157 (5)	0.0180 (6)	0.0180 (6)	0.0006 (4)	0.0066 (5)	−0.0008 (5)
C13	0.0180 (6)	0.0212 (6)	0.0177 (6)	−0.0023 (5)	0.0080 (5)	−0.0019 (5)
C14	0.0286 (7)	0.0278 (7)	0.0238 (7)	0.0021 (5)	0.0134 (6)	−0.0053 (5)
C15	0.0275 (7)	0.0350 (8)	0.0178 (6)	−0.0094 (6)	0.0075 (5)	0.0013 (5)
C16	0.0180 (6)	0.0298 (7)	0.0205 (6)	−0.0023 (5)	0.0061 (5)	−0.0036 (5)
O1WB	0.0291 (12)	0.0366 (15)	0.0210 (11)	0.0019 (10)	0.0126 (9)	0.0000 (10)
O1WA	0.0325 (13)	0.0350 (16)	0.0315 (12)	−0.0090 (11)	0.0204 (10)	−0.0005 (11)
O2W	0.0314 (13)	0.0251 (8)	0.0243 (14)	−0.0066 (7)	0.0193 (11)	−0.0056 (7)

### Geometric parameters (Å, °)

**Table:**

O1—C9	1.3397 (15)	C11—H11A	0.9600
O1—C10	1.4527 (14)	C11—H11B	0.9600
O2—C9	1.2038 (15)	C11—H11C	0.9600
O3—C12	1.2097 (15)	C12—C13	1.5353 (17)
N1—C2	1.3213 (15)	C13—C16	1.5319 (17)
N1—C1	1.3659 (15)	C13—C15	1.5321 (17)
N2—C8	1.3278 (15)	C13—C14	1.5392 (18)
N2—C1	1.3604 (15)	C14—H14A	0.9600
N3—C12	1.3703 (15)	C14—H14B	0.9600
N3—C2	1.3958 (15)	C14—H14C	0.9600
N3—H1N3	0.8462	C15—H15A	0.9600
C1—C5	1.4170 (16)	C15—H15B	0.9600
C2—C3	1.4275 (16)	C15—H15C	0.9600
C3—C4	1.3592 (17)	C16—H16A	0.9600
C3—H3A	0.9300	C16—H16B	0.9600
C4—C5	1.4182 (16)	C16—H16C	0.9600
C4—H4A	0.9300	O1WB—H1WB	0.8550
C5—C6	1.4089 (17)	O1WB—H2WB	0.8586
C6—C7	1.3698 (16)	O1WB—H1WA	0.9712
C6—H6A	0.9300	O1WB—H2WA	0.9888
C7—C8	1.4073 (16)	O1WA—H1WB	0.8984
C7—H7A	0.9300	O1WA—H2WB	0.8078
C8—C9	1.5114 (16)	O1WA—H1WA	0.8576
C10—C11	1.5008 (18)	O1WA—H2WA	0.9243
C10—H10A	0.9700	O2W—O2W^i^	0.739 (3)
C10—H10B	0.9700	O2W—H1W2	0.8319
			
C9—O1—C10	115.85 (9)	C10—C11—H11C	109.5
C2—N1—C1	118.01 (10)	H11A—C11—H11C	109.5
C8—N2—C1	117.02 (10)	H11B—C11—H11C	109.5
C12—N3—C2	129.00 (10)	O3—C12—N3	122.22 (12)
C12—N3—H1N3	117.2	O3—C12—C13	121.68 (11)
C2—N3—H1N3	113.8	N3—C12—C13	116.03 (10)
N2—C1—N1	115.32 (10)	C16—C13—C15	109.37 (11)
N2—C1—C5	122.34 (11)	C16—C13—C12	108.45 (10)
N1—C1—C5	122.34 (10)	C15—C13—C12	112.87 (10)
N1—C2—N3	113.75 (10)	C16—C13—C14	109.33 (10)
N1—C2—C3	123.82 (11)	C15—C13—C14	110.05 (11)
N3—C2—C3	122.44 (10)	C12—C13—C14	106.69 (10)
C4—C3—C2	118.10 (10)	C13—C14—H14A	109.5
C4—C3—H3A	121.0	C13—C14—H14B	109.5
C2—C3—H3A	121.0	H14A—C14—H14B	109.5
C3—C4—C5	120.18 (11)	C13—C14—H14C	109.5
C3—C4—H4A	119.9	H14A—C14—H14C	109.5
C5—C4—H4A	119.9	H14B—C14—H14C	109.5
C6—C5—C1	118.57 (10)	C13—C15—H15A	109.5
C6—C5—C4	123.90 (11)	C13—C15—H15B	109.5
C1—C5—C4	117.52 (11)	H15A—C15—H15B	109.5
C7—C6—C5	118.82 (11)	C13—C15—H15C	109.5
C7—C6—H6A	120.6	H15A—C15—H15C	109.5
C5—C6—H6A	120.6	H15B—C15—H15C	109.5
C6—C7—C8	118.56 (11)	C13—C16—H16A	109.5
C6—C7—H7A	120.7	C13—C16—H16B	109.5
C8—C7—H7A	120.7	H16A—C16—H16B	109.5
N2—C8—C7	124.68 (10)	C13—C16—H16C	109.5
N2—C8—C9	114.68 (10)	H16A—C16—H16C	109.5
C7—C8—C9	120.63 (11)	H16B—C16—H16C	109.5
O2—C9—O1	124.63 (11)	H1WB—O1WB—H2WB	115.4
O2—C9—C8	124.61 (11)	H1WB—O1WB—H1WA	109.0
O1—C9—C8	110.76 (10)	H2WB—O1WB—H1WA	83.0
O1—C10—C11	106.88 (10)	H1WB—O1WB—H2WA	97.1
O1—C10—H10A	110.3	H1WA—O1WB—H2WA	102.7
C11—C10—H10A	110.3	H1WB—O1WA—H2WB	116.1
O1—C10—H10B	110.3	H1WB—O1WA—H1WA	115.9
C11—C10—H10B	110.3	H2WB—O1WA—H1WA	93.6
H10A—C10—H10B	108.6	H1WB—O1WA—H2WA	98.9
C10—C11—H11A	109.5	H1WA—O1WA—H2WA	118.4
C10—C11—H11B	109.5	O2W^i^—O2W—H1W2	81.5
H11A—C11—H11B	109.5		
			
C8—N2—C1—N1	−179.96 (10)	C1—N2—C8—C7	−0.23 (17)
C8—N2—C1—C5	0.51 (16)	C1—N2—C8—C9	−179.34 (10)
C2—N1—C1—N2	−177.43 (10)	C6—C7—C8—N2	0.02 (18)
C2—N1—C1—C5	2.10 (17)	C6—C7—C8—C9	179.08 (10)
C1—N1—C2—N3	178.37 (10)	C10—O1—C9—O2	−0.67 (17)
C1—N1—C2—C3	−1.63 (17)	C10—O1—C9—C8	179.55 (9)
C12—N3—C2—N1	175.30 (12)	N2—C8—C9—O2	3.82 (17)
C12—N3—C2—C3	−4.7 (2)	C7—C8—C9—O2	−175.33 (12)
N1—C2—C3—C4	0.06 (18)	N2—C8—C9—O1	−176.40 (10)
N3—C2—C3—C4	−179.94 (11)	C7—C8—C9—O1	4.45 (15)
C2—C3—C4—C5	1.06 (18)	C9—O1—C10—C11	−172.58 (10)
N2—C1—C5—C6	−0.57 (17)	C2—N3—C12—O3	4.9 (2)
N1—C1—C5—C6	179.93 (10)	C2—N3—C12—C13	−172.27 (12)
N2—C1—C5—C4	178.48 (10)	O3—C12—C13—C16	26.19 (17)
N1—C1—C5—C4	−1.02 (17)	N3—C12—C13—C16	−156.66 (11)
C3—C4—C5—C6	178.40 (12)	O3—C12—C13—C15	147.54 (14)
C3—C4—C5—C1	−0.60 (17)	N3—C12—C13—C15	−35.32 (15)
C1—C5—C6—C7	0.34 (17)	O3—C12—C13—C14	−91.47 (15)
C4—C5—C6—C7	−178.65 (11)	N3—C12—C13—C14	85.68 (13)
C5—C6—C7—C8	−0.08 (17)		

Symmetry codes: (i) −*x*, *y*, −*z*+1/2.

### Hydrogen-bond geometry (Å, °)

**Table:**

*D*—H···*A*	*D*—H	H···*A*	*D*···*A*	*D*—H···*A*
N3—H1N3···O2W	0.85	2.39	3.051 (2)	135
N3—H1N3···O1WB^ii^	0.85	2.40	3.095 (3)	140
O1WB—H2WB···N2	0.86	2.26	3.077 (3)	160
O2W—H1W2···N1^ii^	0.83	2.13	2.948 (2)	167
C3—H3A···O3	0.93	2.23	2.8230 (17)	121

Symmetry codes: (ii) −*x*, *y*, −*z*+1/2.
